# Alignment of Multiple Electrospun Piezoelectric Fiber Bundles Across Serrated Gaps at an Incline: A Method to Generate Textile Strain Sensors

**DOI:** 10.1038/s41598-017-15698-7

**Published:** 2017-11-13

**Authors:** Yu-Hsiang Hsu, Chen-Hao Chan, William C. Tang

**Affiliations:** 1 Institute of Applied Mechanics, National Taiwan University, No. 1, Sec. 4, Roosevelt Rd., Taipei, 106 (R.O.C) Taiwan; 2 Department of Biomedical Engineering, University of California, Irvine, 3120 Natural Sciences II, Irvine, CA 92697-2715 USA

## Abstract

In this paper, we report a new type of electrospinning collector that allows simultaneous collection and alignment of multiple poly(vinylidene fluoride-trifluoroethylene) piezoelectric fiber bundles with a controlled separation. The key enabling feature is the serrated teeth along the edges across an inclined gap as a part of the conductive collector. As a result, the electrical field across the gap is shaped to direct the electrospun fibers to merge into multiple bundles. The sharp points on the serrated teeth provide favorable charge dissipation points and thus fibers are preferentially formed bridging two closest sharp points across the gap. To investigate the effectiveness of serrated teeth on the formation of multiple fiber bundles, three-dimensional finite element simulations are conducted. The corresponding collectors are implemented to experimentally study the resulting electrospun fibers. Both simulation and experimental results suggest that multiple fiber bundles can be formed under the condition of a low teeth pitch to gap distance ratio. Furthermore, a sharper tooth angle results in a higher preferential formation of fiber bundles. Finally, the total electrospinning time should be less than 60 seconds to maintain favorable electric field profile. We also demonstrate that these piezoelectric fiber bundles can serve as ultra-flexible textile sensors.

## Introduction

Electrospinning process is a well-developed and accepted method to create continuous fibers in nano- to micro-scale from a solvent-based solution or a molten solution^1^. Due to its versatility and capability to handle a variety of polymers and to incorporate nanoparticles inside the fibers, it has been widely applied in the fields of environmental filtrations^2,3^, tissue engineering^4–7^, wound healing^8^, drug delivery^9^, fuel cells^10,11^, sensors^12^, power harvesting^13^, etc. Detailed discussions of the applications of electrospinning can be found in review papers reported by Ramakrishna *et al*.^14^ and Bhardwaj and Kundu^15^.

The three basic components to perform electrospinning process are polymer solution, a high electrical potential, and a grounded collector. The dimension, shape, and spatial distribution of electrospun fibers are primarily controlled by three parameters including solution properties, processing setup, and environmental conditions^16–18^. The properties of the electrospinning solution that are crucial to the process include the molecular weight of the polymer and the resultant concentration, viscosity, surface tension and conductivity. In the processing setup, the DC voltage, needle-to-collector distance, and the flow rate of feed solution are important controllable parameters. The electrospinning result is also affected by the temperature and humidity of the processing environment. All of these parameters can dramatically influence the resultant diameter, shape, and spatial distribution of electrospun fibers. In a conventional electrospinning setup, fibers swing and rotate randomly due to the repulsive forces from the induced charges on its surface upon extrusion from the spinneret. Hence, the orientation of collected fibers is usually randomly aligned.

Since the profile and distribution of electrostatic field cannot be altered by the electrospinning solution, processing parameters, and environmental conditions, studies have been conducted to modify the design of collectors to direct the distribution of electrospun fibers. The addition of mechanical rotation to the collector is one of the most common methods. One of the alignment mechanisms is the residual charges on the collected fibers that tend to repel against each other, resulting in alignment pattern. The other is to use a lower and uniform electric field for fiber formation while mechanically pulling the fibers to align on a rotating collector, which can be in the form of a disk^19,20^, a drum^21,22^, or a wire drum^23^. In some cases, a static annular collector with a centrifugal rotating polymer solution jets has also been demonstrated^24^. Fibers can be aligned to form a sheet, a strip, or multiple aligned fibers with random separation distances. However, the level of alignment often starts to deteriorate as the collected fibers thicken due to the gradual accumulation of residual charges on the fiber mat.

Stationary collectors that allow certain degree of alignment of electrospun fibers are also reported, which mainly focus on varying the geometry of stationary collectors to alter electric field distributions to direct fiber alignment. Li *et al*. used two parallel conductive silicon strips separated by a gap to serve as a collector. Driven by electrostatic interactions, the charged nanofibers were stretched to span across the gap to become uniaxially aligned^25,26^. As in a conventional electrospinning setup, the electric field between the spinneret needle and the collector initiates fiber formation along the direction of the electric field lines. However, unlike a conventional setup, the electric field lines in the vicinity of the collector are split into two pointing toward the opposite edges of the gap. As a result, the electrostatic forces acting on the charged nanofibers cause the latter to span across the gap uniaxially. A further improvement of this method is to use an inclined-gap collector proposed and demonstrated by Park and Yang^27^. They modified the configuration of the two strips that formed the collectors so that one strip was oriented horizontally and directly beneath the spinneret needle and the other vertically and offset to the side and below the surface of the first one. These two strips were positioned so that their closest edges formed a gap that was inclined at 45°. Due to the extra height difference compared to the parallel-strip configuration, more space and time were available to allow fibers to be self-aligned. The alignment of the collected fibers suspended in the inclined gap was significantly improved. Following this concept, Xie, *et al*. created radially aligned electrospun fibers by using a point electrode at center of a ring-shape collector^28^. Similarly, orthogonally oriented fibers could be collected by a square collector constructed by two pairs of collecting electrodes orthogonal to each other, where each pair of parallel collectors was grounded alternately for 10 minutes each^29^. Finally, the concept of magnetic electrospinning was also reported, it was based on replacing the two-strip collectors with two grounded magnets with magnetic-particles added into the polymer solutions. As a result, the electrospun fibers were doped with magnetic-particles, and they were stretched to align along the magnetic field across a large gap^30,31^.

Overall, current state-of-the-art electrospinning technology was advanced with the capability to control the alignment of collected fibers resulting in stand-alone fibers, strips, sheets, and mats. Some of the alignment approaches have been adopted as industrial standard process for mass production. However, strategy to direct electrospun fibers to separate into independent and aligned bundles has not been realized. This would be an important technique for small scale applications, such as micro-sensors, textile sensors, and tissue engineering scaffolds. Since electrospun fibers are in the range of nanometer to micrometer, it can be hard to cut or reposition electrospun fibers into multiple parallel aligned fiber bundles after they are formed. In this paper, we present the study on an incline gap with serrated edges as the collector that further improves upon the published inclined-gap collector^27^. The key significance is that the serrated edges across the incline gap enable the ability to focus and direct the electric field at predetermined locations and separations, so that the electrospun fibers aggregate onto the tooth tips and span across the closest opposing tip. This effect was previously verified by two of our experimental studies. First, we experimentally demonstrated that the probability of electrospun fibers collected on a serrated tip could be significantly increased by using a sharp tooth^32^. Second, our experimental findings suggested that using a smaller pitch-to-gap ratio with a sharper tooth angle, multiple aligned fiber bundles with a designed separation could be collected simultaneously^33^.

In this work, this new collector design was used to create uniaxially oriented piezoelectric nanofiber bundles at regular separation intervals. The design, simulation, and experimental findings of three key design parameters of the serrated-edge incline-gap collector for generating multiple P(VDF-TrFE) piezoelectric fiber bundles are presented. Finite-element simulation results on the influence of the serrated edges on the electric field were consistent with the experimental observation with xerographically-cut aluminum film collectors. This work lays the foundation toward achieving ultra-miniaturized piezoelectric transductors for a broad range of sensing and actuating applications at the nano-scale. One of the advantageous applications of these piezoelectric fiber bundles is in ultra-flexible textile sensors for on-body monitoring involving physical movements^34^. The extremely small size and flexibility of the piezoelectric fiber bundles enable wearable sensors that offer superior comfort and sensitivity to conventional sensors. They can be used to monitor arms and legs movements for gait kinematic studies, breathing irregularities to predict onset of asthma attacks, minute body movements for tremor, seizure, or Parkinson disease monitoring, as well as the broad field of sports medicine and health exercise monitoring. As a feasibility study, we demonstrates that the pronation and supination of the forearm, the isometric contraction of brachioradialis, the concentric and eccentric contractions of biceps, and the isometric contraction of biceps can be measured by using a simple setup involving one piezoelectric fiber bundles.

## Results

**Figure 1:**
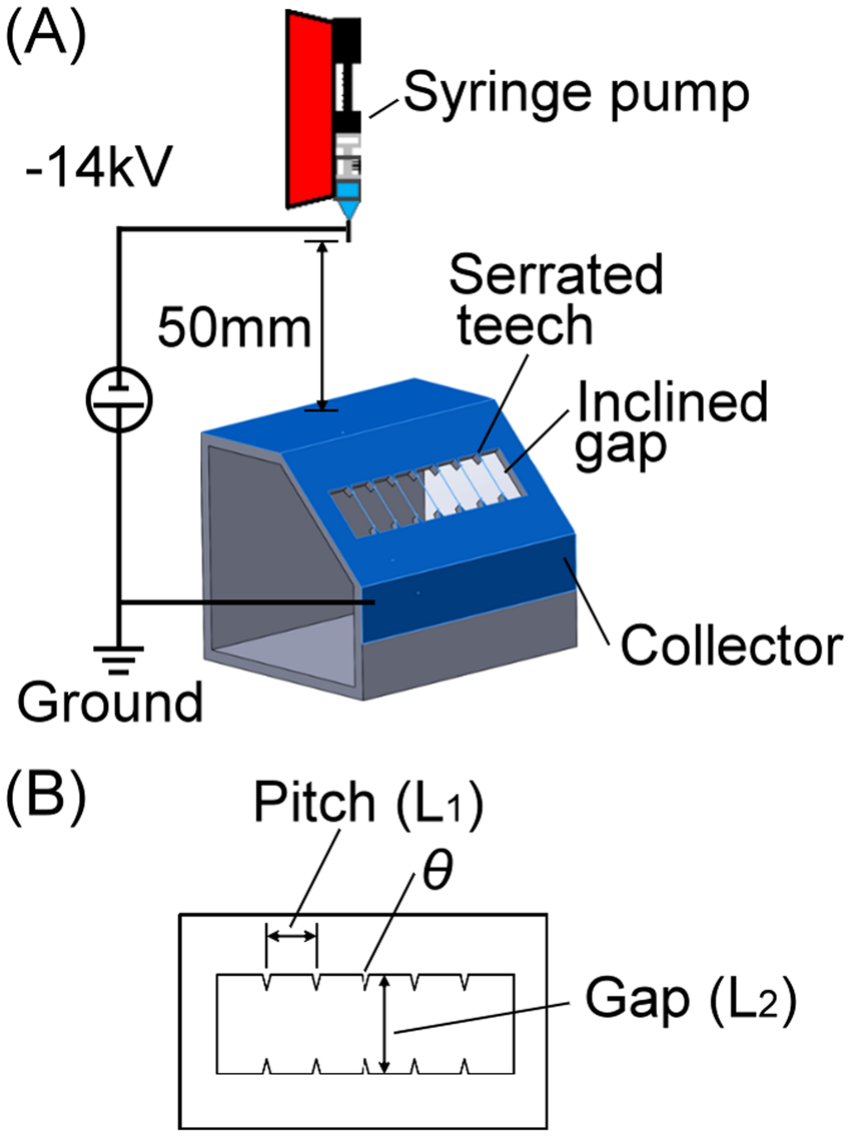
Illustrations of (**A**) the experimental setup of the serrated-edge incline gap collector, (**B**) three design parameters of the serrated teeth.

Figure 1A illustrates the experimental setup of the electrospinning process in this study. The serrated-edge collector was fabricated by xerographically cutting a 5-μm-thick aluminum film attached to a polyvinyl chloride (PVC) tape. The machined aluminum piece was then attached onto a PMMA frame to create a 45° inclined gap. The basic configuration of this collector is shown in Fig. 1B. Three design parameters were studied, including the pitch distance (L1), the gap distance (L2), and the tooth angle θ. In the following sections, these three parameters are presented with [L1, L2, θ] for ease of identifying the design of each version of the serrated-edge collectors. The 20%wt P(VDF-TrFE) (75/25) copolymer solution was continuously fed through a spinneret at a volume flow rate of 0.6 ml/hr at a distance of 50 mm from the top of the collector. A bias voltage of −14 kV was applied at the needle tip with the collector grounded. Electrospinning process duration starting at the formation of the Taylor cone of copolymer solution on the needle tip to stopping the solution delivery and the bias voltage was controlled to achieve the proper amount of fiber bundles.

**Figure 2:**
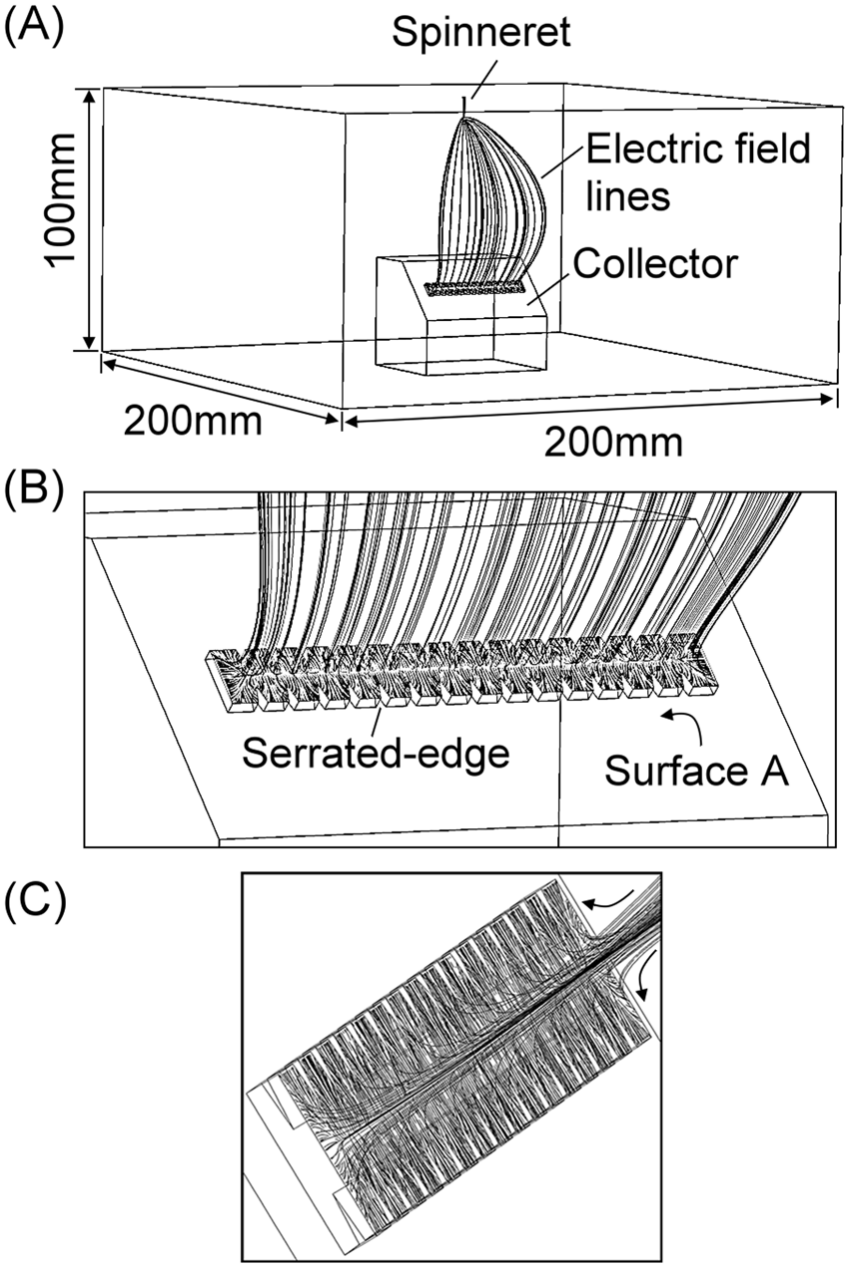
3D finite-element simulation with COMSOL®, (**A**) showing the overall setup and (**B** and **C**) the close-up view of the 3D electric field profile around the serrated-edge incline gap.

Figure 2A shows the 3-D finite element model built in the COMSOL® finite element software, where the designed collector is placed in a 200 mm by 200 mm by 100 mm cuboid box to simulate the surrounding air. Collectors with different design parameters are built to simulate the distribution of electric field and the corresponding electric field lines. Detailed setup of the 3-D finite element simulation is described in the Methods section. Simulated electric field lines that flow from the spinneret to the serrated-edge incline-gap collector are plotted in Fig. 2A with an enlarged image shown in Fig. 2B. These simulated electric field lines represent the most favorable trajectory of electrospun fibers forming across the inclined gap toward the region of the serrated edge. Simulation results showed that electric field lines starting from the spinneret needle tip (not shown) continued toward the central region of the collector and then diverged as they approached the gap, splitting between the upper and the lower sides of the serrated-edge incline gap collector. This effect is shown more clearly from a different perspective in Fig. 2C where arrows are added to show the direction of the electric field lines. This profile of electric field lines is consistent with that reported by Li, *et al*.^25^ and Suk, *et al*.^27^. To study the pattern of collected electrospun fibers, the distributions of electric field in the region of serrated-edge incline-gap on the same plane of surface A (Fig. 2B) were investigated in the following simulation study. The corresponding serrated-edge incline-gap collectors were fabricated and tested to experimentally verify the simulation results. Detailed discussions of polymer solution preparation, collector fabrication, and experimental setup are detailed in the Methods section.

**Figure 3:**
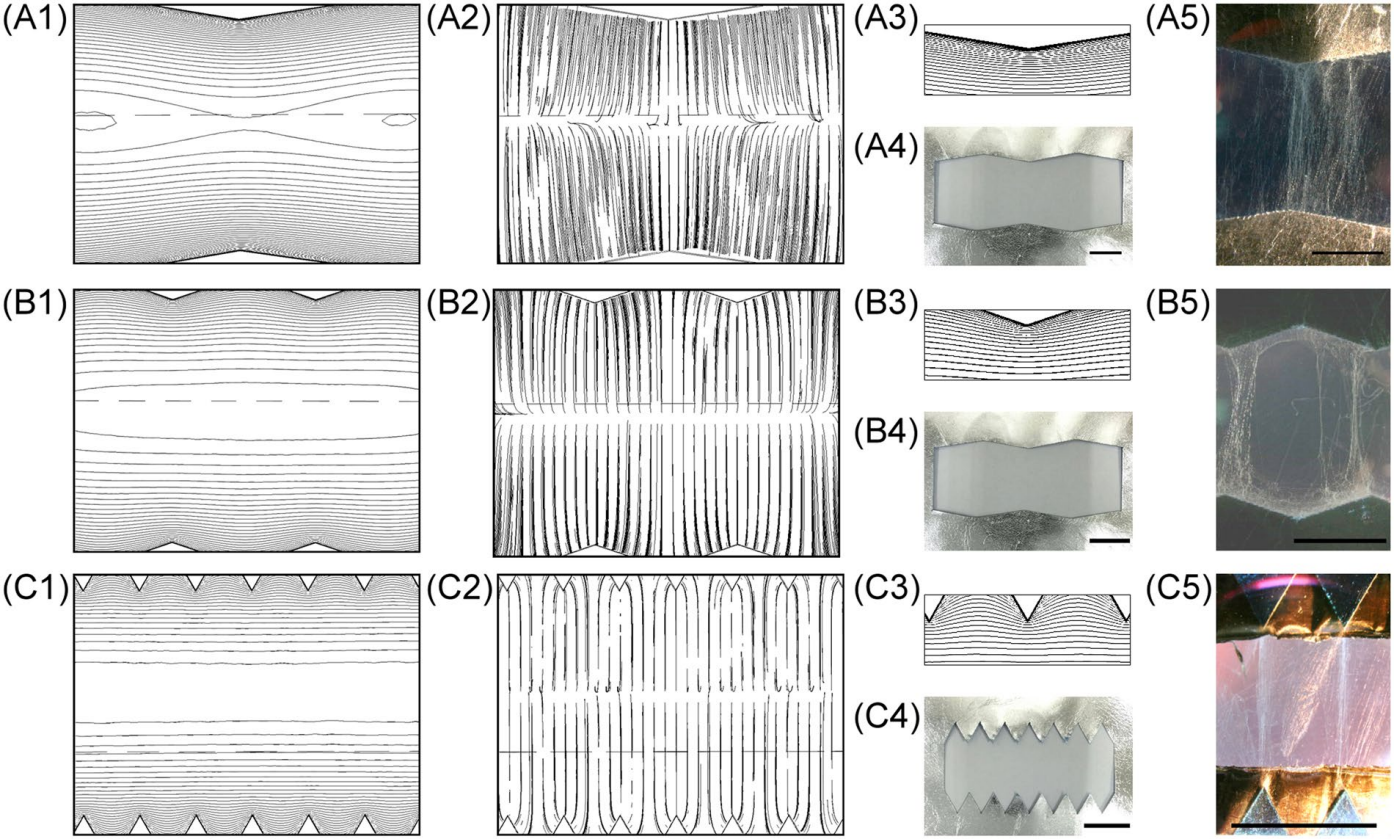
COMSOL® finite-element simulation results, fabricated serrated teeth, and electrospinning results from (**A1**–**A5**) [20 mm, 13.3 mm, 161°]; (**B1**–**B5**) [10 mm, 10 mm, 143°]; and (**C1**–**C5**) [5 mm, 10 mm, 60°] serrated teeth deign; where (**A1**,**B1**,**C1**) are simulated electric potential contours; (**A2**,**B2**,**C2**) are simulated electric field profiles; (**A3**,**B3**,**C3**) are zoomed-in images of electric potential contours near each tooth; (**A4**,**B4**,**C4**) are fabricated serrated teeth; and (**A5**,**B5**,**C5**) are images^32^ of electrospun fibers collected by each collectors. (scale bar = 5 mm).

Figure 3 shows the studies of the effects on the electric field profiles and the electrospinning results of three different tooth angle (θ): 60°, 143°, and 161°. The corresponding electric field simulations, images of the fabricated serrated teeth, and electrospinning results for three versions of the collectors are shown in Fig. 3A1–A5 for [L1, L2, θ] = [20 mm, 13.3 mm, 161°], Fig. 3B1–B5 for [10 mm, 10 mm, 143°], and Fig. 3C1–C5 for [5 mm, 10 mm, 60°], respectively. Figure 3A1,B1 and C1 are the simulated equipotential contours and Fig. 3A2,B2 and C2 are the corresponding electric field lines. The potential difference between equipotential contour lines in Fig. 2A1 are −20 V and −10 V in Fig. 2B1 and C1. The equipotential contour is increasing from negative voltages at central region to 0 volt on the grounded serrated edges. Note that the PMMA frame underneath the serrated-edge incline-gap was the physical structure in the background when both simulation images were generated. The horizontal dashed line was part of the PMMA frame. This dashed line was not part of the simulation results for all the simulation images presented in this paper. Fig. 3A3,  B3 and C3 are the zoomed-in view of the equipotential contours surrounding each tooth. The simulation results suggested that the electric field profile across the gap could be shaped with the serrated edges and that the electric fields were more concentrated on sharper teeth, resulting in better and more intense field alignment between opposing teeth. This provided a more favorable path for the electrospun fibers to span across the gap, where the negatively charged fibers were attracted to the most favorable low-energy state through electrostatic interactions. The simulation results also showed that equipotential contours gradually and rapidly flattened out towards the midline of the collector gap, especially for the version with blunt teeth, which should result in the weakest favorable paths for fiber bundle orientation. These predictions were verified experimentally as shown in Fig. 3A5,  B5 ^32^, and 3C5^32^ showing the absence of preferred orientation of the electrospun fiber bundles between opposing tips with a tooth angle θ of 161° (Fig. 3A5)^32^, which was in sharp contrast to those with θ of 60° (Fig. 3C5) where the great majority of fiber bundles spanned across the gap between opposing tips. The version with θ of 143° showed intermediate results, with a good portion of the fiber bundles exhibiting preferred orientation between opposing tips with some also collected between adjacent teeth (Fig. 3B5). These results demonstrated that the serrated edges in an inclined gap collector could direct electrospun fibers to align at two sharp points of opposing teeth, potentially a powerful tool for controlling electrospun fiber alignment.

**Figure 4:**
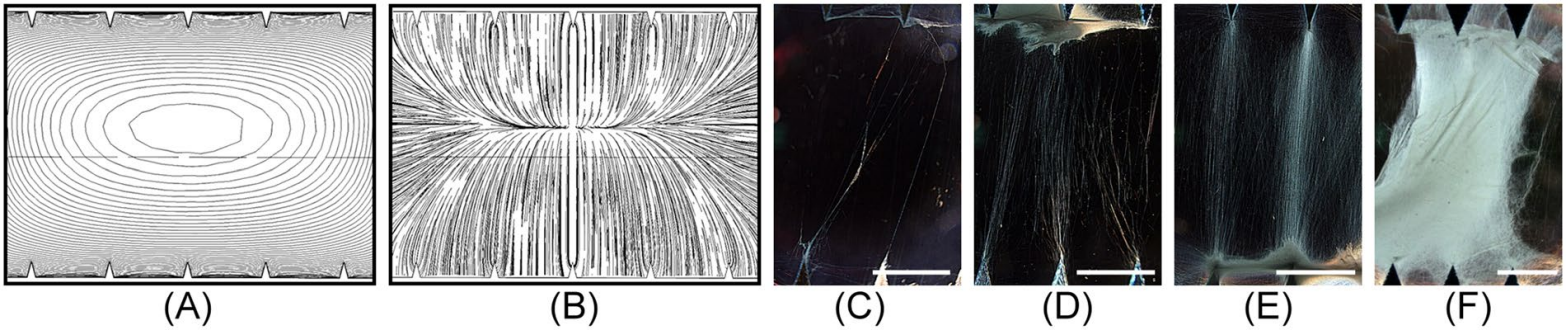
COMSOL® finite-element simulation results of (**A**) electric equipotential contour; and (**B**) electric field profiles of a [5 mm, 15 mm, 30°] collector; and the pattern of collected fibers with electrospinning durations of (**C**) 12 s, (**D**) 30 s, (**E**) 60 s, and (**F**) 300 s^32^. (Scale bar = 5 mm).

A second set of experiments was designed to investigate the effects of electrospinning duration on the resulting fiber bundles. The collector version used in this study was [15 mm, 5 mm, 30°]. Figure 4A and B show the simulated electric equipotential contours and electric field profiles of this design, indicating that the most robust fiber bundle alignment should be between the opposing teeth in the middle of the gap. The potential difference between adjacent equipotential contour lines in Fig. 4A is −5 V, and it is increasing from negative at the central oval line to 0 volt on the serrated edges. Four different electrospinning process durations were studied: 12 s, 30 s, 60 s, and 300 s, with the respective patterns of collected fibers shown in Fig. 4C,D,E and F ^32^. It was found that a longer process duration resulted in not only more electrospun fibers, but also an increase in fibers spanning between teeth not directly opposite to each other. A short processing duration of only 12 s resulted in a small amount of electrospun fibers aligned between tips of opposing teeth (Fig. 4C). On the other hand, a 30 s electrospinning duration resulted in an aggregation of electrospun fibers at the tips of the serrated teeth (Fig. 4D). More fiber bundles were collected diagonally between and around tips when electrospinning duration was increased from 30 s to 60 s (Fig. 4E). With a much longer processing duration at 300 s, the preferred-alignment of electrospun fiber was completely lost, with the collected fibers forming a sheet between the two edges. A reasonable mechanism behind this experimental results could be suggested that the few fibers bundles that were initially collected across the gap occupied the most favorable positions, causing the subsequent incoming fibers to have a less prefer orientation along the tooth tips. This could be due to the accumulation of residual charges on the fibers that alter the discharge pathways of electrostatic energy of the incoming fibers. Therefore, in order to achieve well-aligned and separated fibers, it is important to limit the processing duration, optimally to less than 60 s.

**Figure 5:**
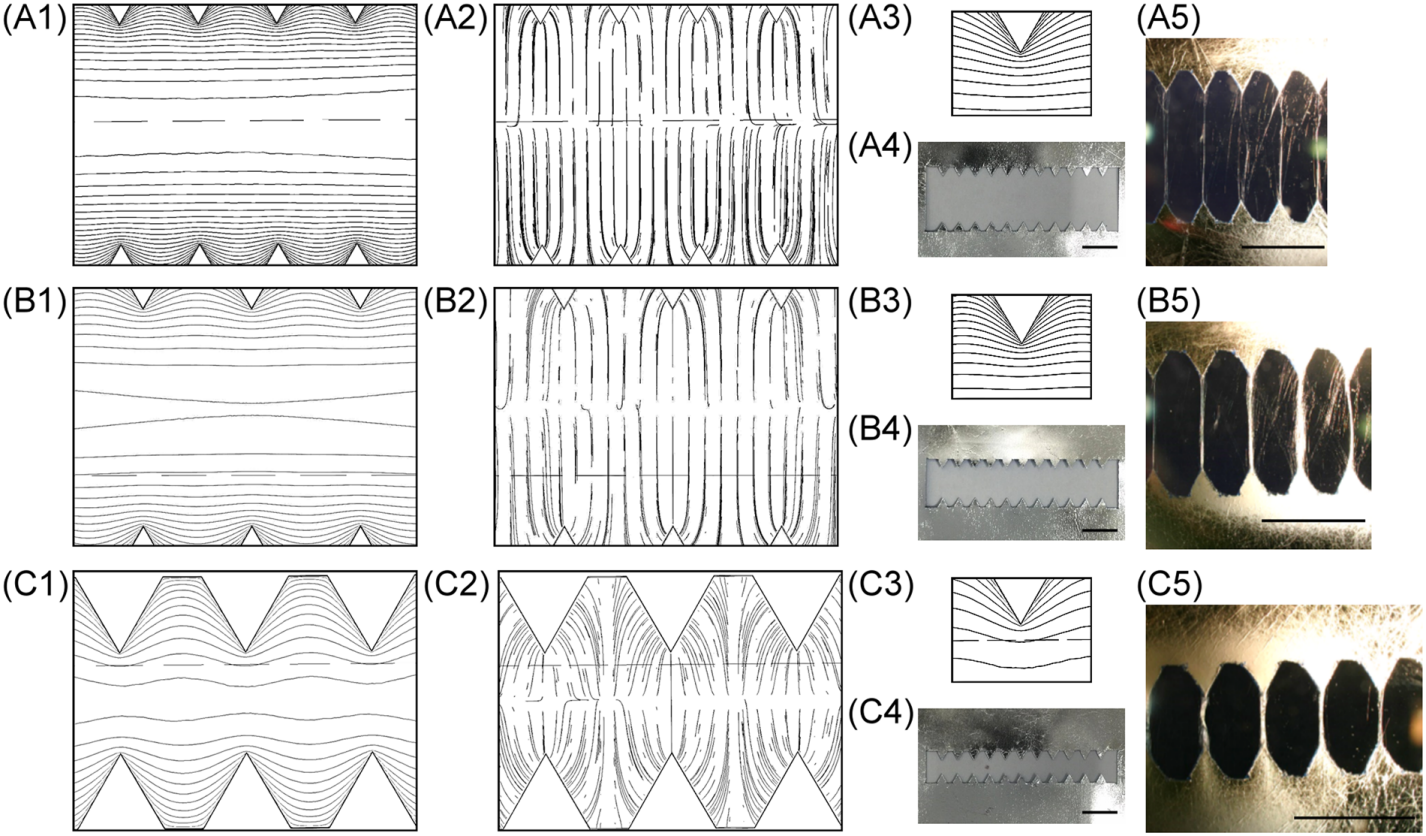
COMSOL® finite-element simulation results, fabricated serrated teeth, and electrospinning results from (**A1**–**A5**) [2.5 mm, 10 mm, 60°]; (**B1**–**B5**) [2.5 mm, 7.5 mm, 60°]; and (**C1**–**C5**) [2.5 mm, 5 mm, 60°] collector deigns; where (**A1**,**B1**,**C1**) are simulated electric potential contour; (**A2**,**B2**,**C2**) are simulated electric field profiles; (**A3**,**B3**,**C3**) are zoomed-in images of electric potential contour near each tooth; (**A4**,**B4**,**C4**) are fabricated serrated teeth; and (**A5**,**B5**,**C5**) are images^33^ (© 2016 IEEE) of fibers collected by each respective collector. (scale bar = 5 mm).

Having experimentally established that sharper tooth angles promote the collection of aligned electrospun fibers at the tooth tips (Figs 3C5 and 4C), collector geometries with θ of 30° and 60° were further studied to investigate the contribution of pitch distance (L1) and gap distance (L2). In this study, the dimension and geometry of the tooth were fixed to investigate the effect of pitch distance and gap distance. Figure 5 shows the simulation results, fabricated serrated edges, and experimental results of collected fibers with θ of 60°. This set of experiment included collectors with L1 fixed at 2.5 mm, and L2 at three different values: 5 mm, 7.5 mm, and 10 mm. The design parameters for Fig. 5A1–A5,B1–B5 and C1–C5 were [2.5 mm, 10 mm, 60°], [2.5 mm, 7.5 mm, 60°], and [2.5 mm, 5 mm, 60°], respectively. The potential difference between adjacent equipotential contour lines was fixed at 5 V for ease of comparison between different designs. The simulation results indicated that electric equipotential contours could be shaped more profoundly with shorter gap distance (Fig. 5A1,B1 and C1). Figure 5C1 shows the highest level of electric equipotential contours shaping in contrast to Fig. 5B1 and A1. The simulated electric field lines of these three gap distances clearly suggested that a much higher probability of fiber alignment between the tips of opposing teeth could be realized by shorter gaps (Fig. 5A2,B2,C2). Figures 5A3,B3 and C3 presented the zoomed-in views of the simulation results. As opposing teeth were moved closer together with a shorter gap distance, the electric equipotential contours were shaped deeper into the gap midline region. To verify the simulation result, the respective collector designs were fabricated and electrospun fibers were collected (Fig. 5A4,B4 and C4). The total electrospinning duration was fixed at 30 s for all experiments in this set of studies. Experimental results are shown in Fig. 5A5 ^33^,B5^33^ and C5^33^, which demonstrated that multiple aligned fiber bundles were successfully collected spanning between the tips of opposing teeth, and that the shortest gap distance of 5 mm exhibited the best results of multiple fiber bundle alignment with the fewest misaligned fibers (Fig. 5C5).

**Figure 6:**
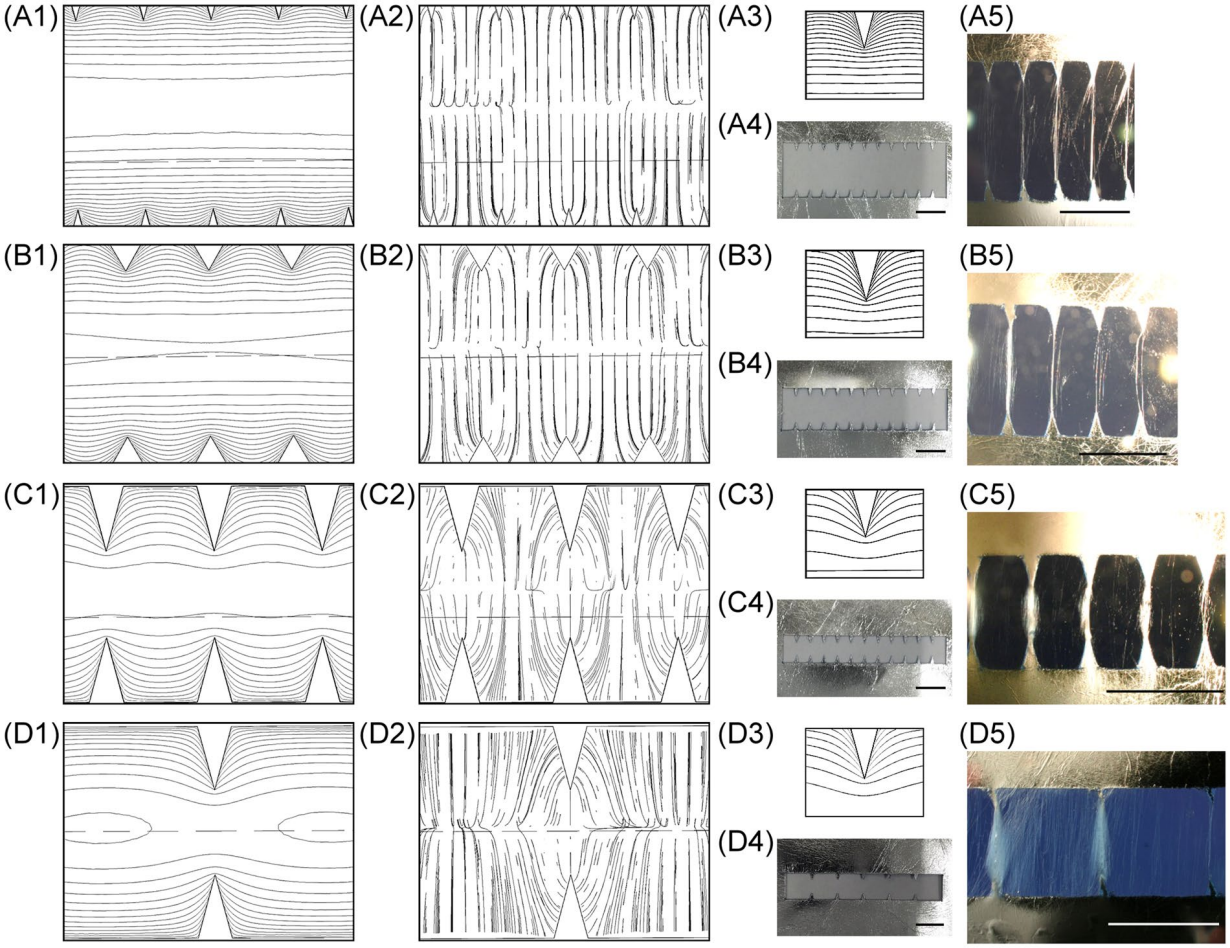
COMSOL® finite-element simulation results, fabricated serrated teeth, and electrospinning results from (**A1–A5**) [2.5 mm, 10 mm, 30°]; (**B1–B5**) [2.5 mm, 7.5 mm, 60°]; (**C1–C5**) [2.5 mm, 5 mm, 30°]; and (**D1–D5**) [5 mm, 5 mm, 30°] serrated teeth deign; where (**A1**,**B1**,**C1**,**D1**) are simulated electric potential contours; (**A2**,**B2**,**C2**,**D2**) are simulated electric field profiles; (**A3**,**B3**,**C3**,**D3**) are zoomed-in images of electric potential contours near each tooth; (**A4**,**B4**,**C4**,**D4**) are fabricated serrated teeth; and (**A5**,**B5**,**C5**,**D5**) are images^33^ (© 2016 IEEE) of electrospun fibers collected by each respective collector. (scale bar = 5 mm).

This set of studies of varying the gap distance was repeated on collectors with 30° tooth angle. Figure 6A1–A5,B1–B5 and C1–C5 show the simulated electric equipotential contours, electric field profiles, fabricated collectors, and experimental results^33^. Similarly, L1 was held constant at 2.5 mm while L2 was changed with three different values: [2.5 mm, 10 mm, 30°], [2.5 mm, 7.5 mm, 30°], and [2.5 mm, 5 mm, 30°]. Electrospinning duration was likewise fixed at 30 s for comparison. Simulated equipotential contours are shown in Fig. 6A1,B1 and C1, and the potential difference between adjacent equipotential contour lines was also fixed at 5 V. The corresponding zoomed-in views of the equipotential contours around the tips are shown in Figure 6A3,B3 and C3. These simulation results are consistent with those from collectors with 60° tooth angle: the shortest gap distance of 5 mm exhibited the deepest electric equipotential contour shaping, reaching the gap midline region. Similarly, the simulated electric field profiles (Fig. 6A2,B2, and C2) also suggested the highest likelihood of electrospun fiber alignment between directly opposing tips when the gap is the shortest. Experimental results were similarly in excellent agreement with the simulated predictive model.

The effects of pitch distance (L1) could be analyzed by comparing the simulation and experimental results from [5 mm, 10 mm, 60°] and [2.5 mm, 10 mm, 60°] collectors shown in Figs 3C1–C5 and 5A1–A5, respectively. A comparison of the two experimental results shown in Figs 3C5 ^33^ and 5A5 ^33^ indicated that there was less fiber misalignment with a shorter pitch distance (L1). Similarly, a comparison of the experimental results from the [2.5 mm, 5 mm, 30°] and [5 mm, 5 mm, 30°] collectors shown in Fig. 6C1–C5 and D1–D5, respectively, indicated consistently that shorter L1 was better in avoiding fiber misalignment^33^. A comparison of the simulated electric equipotential contours shown in Fig. 6C1 and D1 demonstrates that the electric equipotential contour shaping was more extensive throughout the entire gap with a smaller pitch distance even though the level of field shaping around the tip region was similar (Fig. 6C3 and D3). This analysis suggests that the more extensively shaped electric field profile with densely spaced serrate teeth performs better in aligning fibers than the less shaped one from sparsely spaced teeth. As a result, much fewer electrospun fibers were collected between opposing flat edges on the collector with shorter L1, as evidenced when comparing Fig. 6C5 ^33^ and D5.

In summary, to create fiber bundles with maximum alignment, both pitch distance (L1) and gap distance (L2) should be shortened, with the best design having L1 < L2. Furthermore, both the simulation and experimental results suggested that the trajectory of electrospun fibers likely followed the path of the electric field lines. Fibers would first enter the gap in the central portion, then immediately diverge toward the two sides of the grounded serrated edges. The level of electric field shaping near the central region, therefore, played a crucial role in the aggregation and alignment of electrospun fibers between opposing teeth. The conditions of increased parallel equipotential contour lines at the central portion of the gap region could result in more fibers aligned between the two edges in parallel, similar to the original incline-gap collector. On the other hand, if the gap distance and pitch distance were decreased, the level of electric field distortion and shaping at the central region would become more intense. Under this condition, the entering trajectory of fibers at the central region would be altered accordingly, and fibers would be more intensely diverted to span across two nearest tips from opposite edges with serrated teeth.

**Figure 7:**
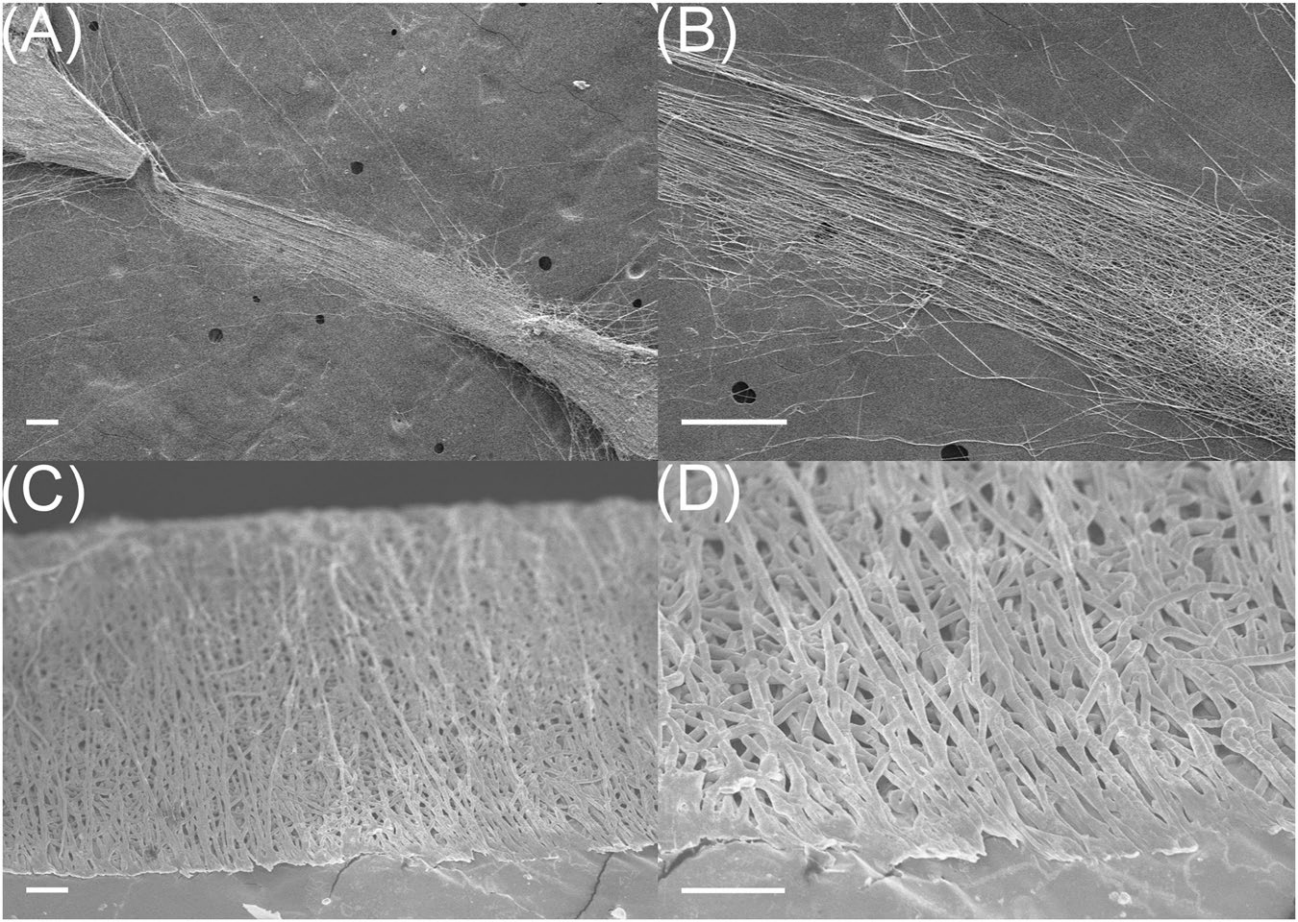
SEM micrographs of (**A** and **B**) collected fiber bundles from a [2.5 mm, 5 mm, 30°] collector; and (**C** and **D**) cross-sectional micrographs of a fiber bundle. The scale bars are (**A** and **B**) 2 mm, (**C**) 200 μm, and (**D**) 20 μm.

Figure 7 shows the SEM micrographs of P(VDF-TrFE) fiber bundles collected with the [2.5 mm, 5 mm, 30°] collector. The electrospinning duration was 40 seconds. The aggregation of electrospun fibers at the tips of the two opposing teeth can be clearly observed in Fig. 7A. The location of the fiber entry point at the center of the gap can also be observed on the zoomed-in image shown in Fig. 7B, which was predicted with the simulated electric field profiles. The measured width of the fiber bundle was 369 μm. To expose and examine the cross-sectional features of the fiber bundles, they were frozen in liquid nitrogen and broken in half. Figure 7C and D show two of SEM images in different magnifications. It was found that electrospun fibers from our processing approach were stacked and overlapped to form a bundle. The average diameter of one strand of fiber was 1.0 μm with a standard deviation of 0.2 μm.

**Figure 8:**
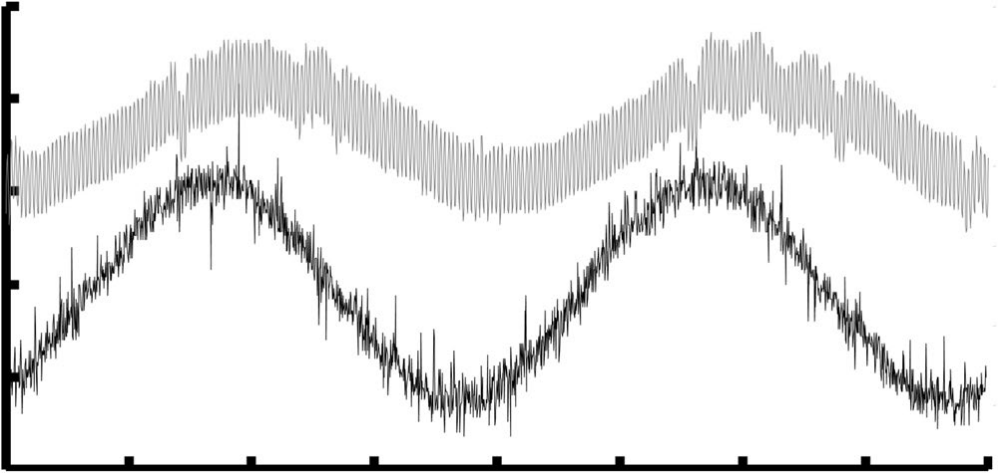
Time domain response of the piezoelectric P(VDF-TrFE) fiber bundle at 0.5 Hz. Gray – output of the piezoelectric fiber bundle and Black – drive voltage of the shaker.

Since the electrospinning process was performed under a high DC bias voltage, the P(VDF-TrFE) fibers were simultaneously poled along the axial direction during the process. Each collected P(VDF-TrFE) fiber bundle can be used directly as a piezoelectric sensor without the need for standard annealing and poling steps. To test the piezoelectric performance, one of the piezoelectric fiber bundles was extracted from the [2.5 mm, 5 mm, 60°] collector by carefully cutting the aluminum collector tips where both ends of the bundle were attached, and fixing them on a suspension on top of a shaker. The fiber bundle was stretched when attaching to the suspension to create a constant tension within the fibers. A charge amplifier (Kistler, Type5006) and a low-pass filter with corner frequency at 10 Hz were used in series with an oscilloscope to measure the charge displacement in the fiber bundle. The vibration frequency of the shaker was set at 0.5 Hz while signals were monitored and recorded with a digital oscilloscope (Tektronix, MDO 3014). Figure 8 shows one of the experimental results of using the fiber bundle as a vibration sensor. Black and gray lines are signal of the shaker driving voltage and the signal of the piezoelectric fiber bundle, respectively, indicating good responses from a very simple experimental setup under room temperature and atmosphere.

**Figure 9:**
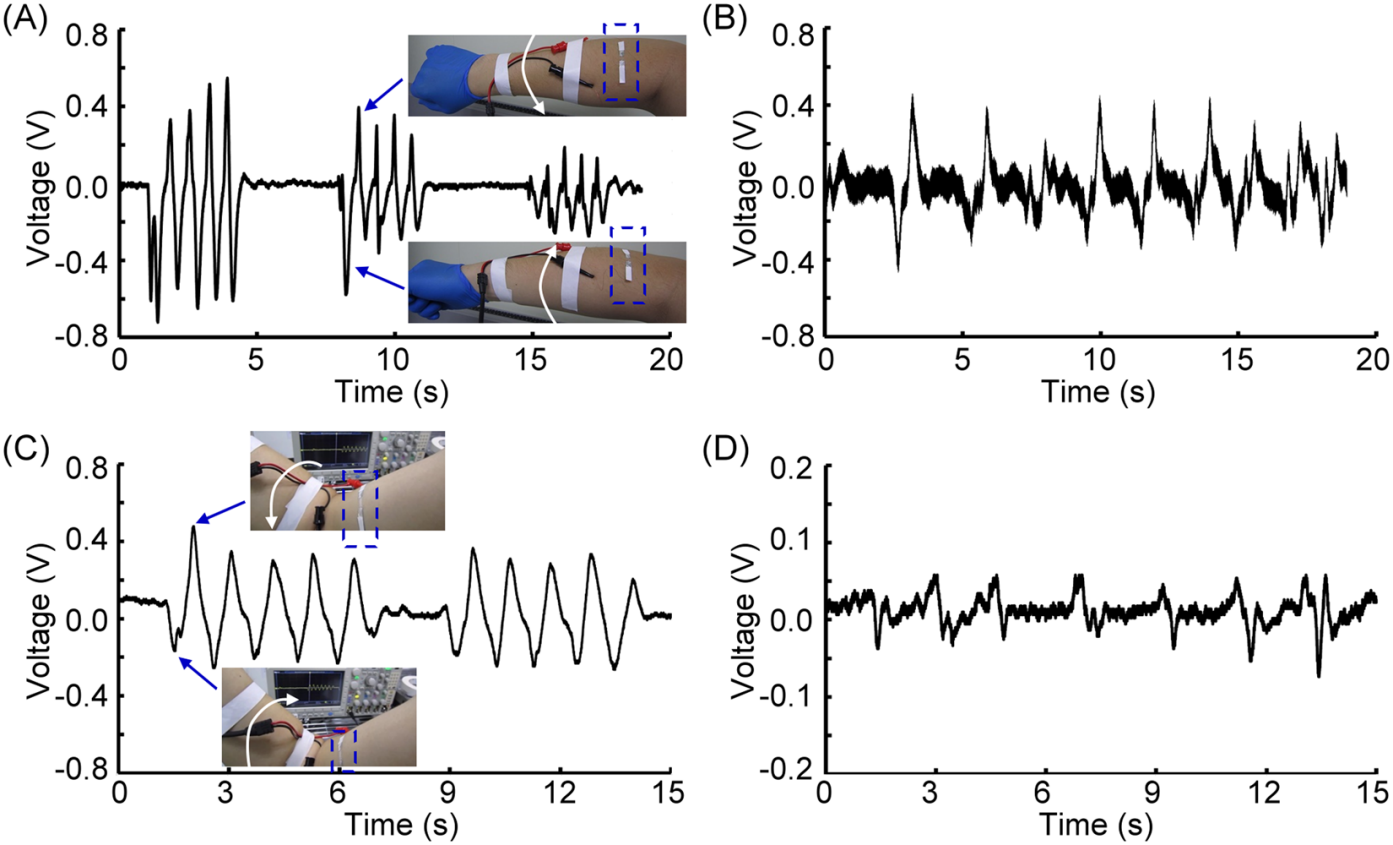
Time trace signals measured by the piezoelectric fiber bundles as textile sensor, they are measurements of the pronation and supination of the forearm (**A**), the isometric contraction of brachioradialis (**B**), the concentric and eccentric contractions of biceps (**C**), and the isometric contraction of biceps (**D**).

Having the capability to create piezoelectric fiber bundles, we conducted a series of experiments to study the feasibility of using these bundles as the key component of a wearable textile sensors to monitor human body movements. We first attached the fiber bundle array onto a soft 3 M™ double coated tissue tape, then cut out one piezoelectric fiber bundle from the collector followed by attaching it perpendicularly to the brachioradialis of the forearm and the biceps of the upper arm as shown in the insets of Fig. 9A and C (highlighted by dashed boxes), respectively. A current amplifier (Standford Research Systems, SR570) with a low pass filter with corner frequency at 10 Hz was used as the interface circuit. Figure 9 shows the experimental results, including measurements of the pronation and supination of the forearm (Fig. 9A), the isometric contraction of brachioradialis (Fig. 9B), the concentric and eccentric contractions of biceps (Fig. 9C), and the isometric contraction of biceps (Fig. 9D). The sensitivity of current amplifier was set at 500 pA/V when the experimental results in Fig. 9A and C were collected, and 200 pA/V for the ones shown in Fig. 9B and D. The first sets of experiments was to measure the circumference variation of brachioradialis of the right forearm during pronation and supination contractions as shown in Fig. 9A. Three different levels of pronation and supination of forearm were tested. These three different levels of contractions were successfully detected, where it was from the higher one on the left to the lower on the right. Furthermore, the piezoelectric bundle was sensitive enough to detect the direction of rotations, where supination of forearm generated negative voltages and pronation generated positive voltages. The supplementary video (Fig. 9A.avi) shows the real-time recording of this experiment. Figure 9B shows the experimental result of the isometric contraction of brachioradialis, where the forearm was not moving but the level of contraction was increased. This experimental result demonstrated that the isometric contraction could be readily detected by the piezoelectric fiber bundle. The contraction or relaxation of brachioradialis can be inferred from the decreasing or increasing output voltage. The supplementary video (Fig. 9B.avi) shows the recording of this experiment.

Similar experiments were also conducted on the right biceps. Figure 9C shows the measurement of concentric and eccentric contractions of the biceps, where the circumferential variations of biceps were monitored. These experimental findings again demonstrated the sensitivity of the piezoelectric fiber bundles. The concentric contractions increased the biceps diameter, resulting in a decreasing negative voltage. On the other hand, eccentric contraction decreased the diameter of biceps, and it generated an increasing voltage pattern. Finally, the isometric contraction of biceps was also studied and is shown in Fig. 9D. A different pattern of contractions from the biceps was measured. It also demonstrated that during muscle contraction, a negative voltage was generated. Conversely, a positive voltage was generated during muscle relaxation. In summary, these experimental studies demonstrated the feasibility of applying the electrospun piezoelectric fiber bundles to enable wearable textile sensors. Supplementary videos show two of the experiments conducted for studying the concentric and eccentric contractions of biceps (Fig. 9C.avi) and its isometric contractions (Fig. 9D.avi).

## Conclusion

In this paper, a new type of electrospinning collector was reported for simultaneously generating multiple piezoelectric fiber bundles with controllable separation and alignment. The key novelty of this collector was the addition of optimally designed serrated teeth to the edges of an inclined gap collector to shape the electric potential contours inside the gap. The net effect was to create an electrostatically favorable path between every pair of directly opposing teeth for the negatively charged fibers to align and settle onto a low-energy state. These sharp points facilitated the relaxation of residual charges and promoted the aggregation of the electrospun fibers. To identify the optimal design parameters of the serrated teeth, the pitch distance, gap distance, tooth angle, and process duration were studied and analyzed. Both simulation and experimental results were in excellent agreement that serrated teeth with a sharp tooth angle, a short pitch distance and a short gap distance resulted in multiple independent fiber bundles. Experimental findings suggested these optimal parameters: 30° tooth angle (θ), 2.5 mm pitch distance (L1), 5 mm gap distance (L2), and less than 60 s of electrospinning duration. Also, the condition of L1 < L2 could further facilitate the formation of fiber bundles while minimizing the probability fiber formation spanning across different opposing teeth and adjacent teeth. SEM micrographs demonstrated that P(VDF-TrFE) fibers, each about 1 µm in diameter, were formed preferentially and aggregated at the tooth tips. These results verify that shaping the electric potential contour in the gap region by introducing sharp teeth could facilitate the formation of fiber bundles with a minimal interference from adjacent bundles. Finally, it was demonstrated that the electrospun P(VDF-TrFE) fiber bundle possessed good piezoelectric properties as fabricated without the need for further poling and annealing. A low frequency vibration at 0.5 Hz could be measured with a simple setup using one fiber bundle. The high sensitivity at low frequency range was due to the small diameter of the P(VDF-TrFE) fibers at around 1 µm. It could serve as a very flexible low-frequency mechanical piezoelectric sensor. We have demonstrated its capability with measuring the periodic contraction profile of both brachioradialis and biceps. With further development, this method could offer a simple and straightforward approach to manufacture piezoelectric fiber bundles with enhanced piezoelectric performance for use in many micro- and nano-scale sensor applications.

## Methods

### Fabrication process of the serrated-edge incline gap collector

A 165 μm thick polyvinyl chloride (PVC) tape was placed on a computer-controlled xerographic cutting machine (Graphtech Corp.). Residual electrostatic charges of the PVC tape were removed with an ionizing blower. A 5 μm thick aluminum foil was then attached onto the surface of the PVC tape. The serrated teeth and the gap region were machined out and the unwanted materials peeled off. The finished aluminum/PVC pattern was then removed and attached to a 2-mm-thick PMMA frame to create a 45° incline gap.

### Preparation of the P(VDF-TrFE) (75/25) copolymer solution

The electrospinning solution was prepared from P(VDF-TrFE) powder in 75/25 ratio (KUREHA, Japan). The solvent was prepared by mixing N,N-dimethyl aceramide (DMAc) (Acros Organic Corp.) and methylethyl ketone (MEK) in 25 to 75 volume ration (25/75 v/v). The P(VDF-TrFE) powder was added into the solvent to reach 20% by weight. This mixture was stirred overnight before electrospinning experiment.

### Experimental setup of the electrospinning process

The P(VDF-TrFE) electrospinning solution was loaded into a glass syringe and placed on a syringe pump, which was secured vertically on top of the serrated-edge incline gap collector at a 50 mm distance as shown in Fig. 1A. A 31-gauge stainless steel dispensing blunt needle with polished tip was used as the spinneret and installed on the syringe. A DC bias voltage of −14 kV from a high-voltage DC power supply (Matsusada Precision Inc.) was applied on the needle tip with the collector set to ground. The electrospinning process was initiated by first turning on the DC bias voltage for 10 s followed by turning on the syringe pump to introduce the electrospinning solution at a volume flow rate of 0.6 ml/hr. As static charges started to accumulate on the surface of the emerging droplet at the needle tip, a Taylor cone was formed and the electrospinning process was initiated. The collected fibers were then studied under an inverted microscope (Olympus, ×71) and Scanning Electron Microscope (Hitachi High Technologies America, Inc., S-4800).

### Experimental setup of textile sensor

The collected piezoelectric fiber bundles and the aluminum/PVC collector was first attached to a soft 3 M™ double coated tissue tape followed by cut out one of the piezoelectric fiber bundle. Then, it was attached to the skin of the brachioradialis and biceps by the adhesive soft 3 M™ double coated tissue tape. The two aluminum electrodes connected on two ends of the piezoelectric bundle were connected to a current amplifier (Standford Research Systems, SR570) with a low-pass filter with corner frequency at 10 Hz in series. The detected signal picked up by the piezoelectric bundle was monitored and recorded with a digital oscilloscope (Tektronix, MDO3014).

### Finite element simulation

Different versions of the 3D models of the serrated-edge incline gap collector were built with Solidworks® and imported into COMSOL® Multiphysics Simulator as shown in Fig. 2A. The collector was placed at the center-bottom of a 200 mm by 200 mm by 100 mm cuboid box. The cuboid box was used to represent the surrounding air. The serrated-edge incline gap collector was simulated by placing a layer of aluminum sheet on a 45° inclined frame. A small tube was placed 50 mm above the upper flat surface of the collector to simulate the spinneret. Electrostatic Model within COMSOL® was used in this study. Relative permittivity of 1 and conservation of charge were set in the cuboid box to simulate the electric field in the air during electrospinning process. The boundary condition of zero charge was set on the edges of the surrounding air and the collector frame. The electric potential of spinneret was set at −14 kV and the aluminum collector was electrically grounded. The initial condition of this model was 0 volt. Finally, stationary study was conducted in each design for steady-state simulation. The steady state results of both electric equipotential contours and electric field profiles of different designs in the gap region on the same plane shown in Fig. 2A were generated for comparison.

## Electronic supplementary material

Figure 9A.avi

Figure 9B.avi

Figure 9C.avi

Figure 9D.avi
